# Small Extracellular Vesicles and Their Involvement in Cancer Resistance: An Up-to-Date Review

**DOI:** 10.3390/cells11182913

**Published:** 2022-09-17

**Authors:** Artur Słomka, Miroslaw Kornek, William C. Cho

**Affiliations:** 1 Department of Pathophysiology, Nicolaus Copernicus University in Toruń, Ludwik Rydygier Collegium Medicum in Bydgoszcz, 85-094 Bydgoszcz, Poland; 2 Department of Internal Medicine I, University Hospital of the Rheinische Friedrich-Wilhelms-University, 53127 Bonn, Germany; 3 Department of Clinical Oncology, Queen Elizabeth Hospital, Kowloon, Hong Kong, China

**Keywords:** small extracellular vesicles, exosomes, chemoresistance, non-coding RNAs, circular RNAs

## Abstract

In recent years, tremendous progress has been made in understanding the roles of extracellular vesicles (EVs) in cancer. Thanks to advancements in molecular biology, it has been found that the fraction of EVs called exosomes or small EVs (sEVs) modulates the sensitivity of cancer cells to chemotherapeutic agents by delivering molecularly active non-coding RNAs (ncRNAs). An in-depth analysis shows that two main molecular mechanisms are involved in exosomal modified chemoresistance: (1) translational repression of anti-oncogenes by exosomal microRNAs (miRs) and (2) lack of translational repression of oncogenes by sponging of miRs through long non-coding RNAs (lncRNAs) and circular RNAs (circRNAs). At the cellular level, these processes increase the proliferation and survival of cancer cells and improve their ability to metastasize and resist apoptosis. In addition, studies in animal models have shown enhancing tumor size under the influence of exosomal ncRNAs. Ultimately, exosomal ncRNAs are responsible for clinically significant chemotherapy failures in patients with different types of cancer. Preliminary data have also revealed that exosomal ncRNAs can overcome chemotherapeutic agent resistance, but the results are thoroughly fragmented. This review presents how exosomes modulate the response of cancer cells to chemotherapeutic agents. Understanding how exosomes interfere with chemoresistance may become a milestone in developing new therapeutic options, but more data are still required.

## 1. Prima Facie of Extracellular Vesicles (EVs) in Cancer

In recent decades, epoch-making advances in biomedical sciences have made it possible for us to understand the role of extracellular vesicles (EVs) in the development and progression of cancer. This is mainly due to the fact that EVs are a universal envoy of biological information, and they are able to modulate the cellular phenotype, often changing the properties of cells diametrically [1,2]. As reflected in the stunning number of publications on the topic, tremendous progress has been made in understanding the role of EVs in neoplasia since their first description in 1946 [3,4].

The peculiarity of these structures in tumor formation and evolution may be considered on multiple levels. EVs are crucial spinning wheels in tumor disease machinery. Still, to consider these roles more distinctly, they can be viewed in a biological and clinical context, which is briefly outlined below. The first point of view is about how EVs, through the transport of biological information from cell to cell, modulate tumor formation and progression. The second point of view is the clinical use of EVs as non-invasive markers in cancer diagnosis, prediction of patient outcomes, and treatment response.

EVs are at the forefront of controlling virtually every stage of carcinogenesis [5]. The indicated phenomenon is possible due to the abundance of EVs and their diversity of surface protein markers and cargo of non-coding ribonucleic acids (ncRNAs) [6]. Therefore, EVs are structures that are released by tumor cells and tumor-associated cells to create an optimal environment for tumor growth, survival, and metastasis [7,8,9]. Out of all EV populations, exosomes are especially known to be involved in modulating the resistance of cancer cells to chemotherapy and radiotherapy [10,11].

From a clinical perspective, EVs can be used as appropriate biomarkers to evaluate cancer patient evolution. Many studies have indicated that EVs with high sensitivity and specificity can be used as liquid biopsies in the diagnosis and prognosis of patients’ outcomes and responses to treatment [12,13,14,15]. Additionally, meta-analyses, a powerful statistical tool, have confirmed the clinical utility of EVs for cancer detection and prognoses [16,17,18,19]. Let us say, then, that EVs are cancer detection’s ‘ne plus ultra’. That is, a great deal of scientific effort has been made to prove the clinical utility of EVs in cancer diagnosis, prognosis, and treatment response assessment. However, we must find tools to simplify and make inexpensive a process for the robust detection of EVs in body fluids that can be used in everyday clinical practice. As the later part of our manuscript shows, modulation of the action of EVs can also reduce or eliminate resistance to chemotherapeutic agents, a problem seen as the bane of modern oncology.

## 2. Prima Facie of Extracellular Vesicles (EVs) in Chemoresistance

Despite significant progress in systemic cancer treatment, chemotherapy continues to be one of the mainstays of therapy for many types of cancer [20]. However, the effectiveness of chemotherapy is significantly limited by the partial or total insensitivity of neoplastic cells to cytotoxic drugs. This common phenomenon is referred to as chemoresistance, and it is estimated to be responsible for treatment failure and for the deaths of over 90% of patients with cancer [21,22]. This alarming number urges researchers to understand the mechanisms of drug resistance, as combating it is undeniably crucial to successful treatment. Basically, two forms of resistance to chemotherapy have been described, namely intrinsic resistance, where cancer cells have natural drug resistance, and acquired resistance, which is developed by cancer cells through various molecular mechanisms. These sophisticated machineries include, among others, active drug efflux, drug inactivation or changing of the drug target point, forceful DNA repair, and proautophagic, as well as antiapoptotic activities [23,24]. Currently, the literature emphasizes that EVs are the cardinal modulators of chemoresistance, which has been confirmed in numerous experimental and clinical studies [25,26,27,28]. Understanding the role of EVs in chemoresistance is therefore key to explaining the mechanisms of the phenomenon. It is also of great practical importance in oncological treatment—the use of appropriate EV inhibitors may improve the effectiveness of anticancer therapy [29]. This review describes the importance of EVs for regulating cancer cell responses to chemotherapy by focusing on the most up-to-date and clinically quintessential relationships between EVs and chemoresistance. As our team, a little while back, noted, most studies have identified the centrality of the role of exosomal ncRNAs in chemoresistance [30]. Hence, we present here the mechanisms by which these types of EVs modulate the response to cytotoxic drugs by microRNAs (miRNAs, miRs), long non-coding RNAs (lncRNAs), and circular RNAs (circRNAs), all of which have a cardinal role in the regulation of gene expression [31,32,33]. Studies, described in detail below, have shown that the primary mechanism of exosome involvement in chemoresistance is the transport of nucleic acid cargoes from drug-resistant to drug-sensitive cancer cells, thereby enabling the latter to acquire resistance to treatment. Other non-cancerous cells or tumor microenvironment (TME) cells, including cancer-associated fibroblasts (CAFs) and tumor-associated macrophages (TAMs), can induce chemoresistance to cancer via exosomes [25]. Reasoning from this fact, the studies presented in this review have shown that exosomes may likewise be involved in the sensitization of cancer cells to cytotoxic drugs. These opposite properties of exosomes, induction, or reduction of chemoresistance, are determined by their cargo during intercellular communication [30]. Moreover, exosomes not only modulate the response to cytotoxic therapy but can also induce radioresistance [34].

## 3. Exosomes as Chemoresistance Mediators

The biological properties of exosomes as drivers of chemotherapy resistance have been confirmed in numerous in vitro and in vivo studies. The most common form of this phenomenon is multidrug resistance (MDR) [24], which can be described as “purchased” cancer cells resistant to cytotoxic drugs with different chemical structures and modes of action [35]. Below, we present the role of exosomal ncRNAs in these processes, depending on the drug used in cancer therapy. If such data are available, we mechanistically explain how exosomal ncRNAs modulate the response of cancer cells to a specific chemotherapeutic agent.

### 3.1. Resistance to Platinum-Based Therapy

The literature review indicates that three forms of exosomal ncRNAs are involved in developing cancer cells’ resistance to cisplatin (cis-diamminedichloridoplatinum, DDP): miR [36,37], lncRNA [38,39,40,41,42], and circRNA [43,44,45,46,47,48,49]. Details of the involvement of specific ncRNA forms in the development of cisplatin resistance are provided below.

Shi et al. showed that exosomal miR-193 promotes cisplatin chemoresistance in esophageal cancer cells [36]. The authors used two types of esophageal cancer cell lines in their experiment, namely the TE-1 line, which is sensitive to cisplatin, and the TE-1/DDP line, which is resistant to this drug. They determined that miR-193 in exosomes released from TE-1/DDP can be transferred to TE-1, rendering the latter line resistant to cisplatin. This phenomenon was associated with the silencing of the transcription factor AP-2 gamma (TFAP2C) by miR-193, resulting in a lack of cisplatin-induced cell cycle arrest and apoptosis [36]. Not only do cancer cells have the ability to release miR-rich exosomes; more recently, Zhang et al. demonstrated that CAF-derived exosomes confer cisplatin resistance in non-small cell lung cancer (NSCLC) cells via the transport of miR-130a [37]. NSCLC cells take up miR-130a-rich exosomes produced by cisplatin-resistant CAFs, which promotes the NSCLC cell’s survival rate [37]. Interestingly, the authors found that a specific RNA-binding protein, pumilio homolog 2 (PUM2), is responsible for packaging miR-130a into exosomes [37]. Cisplatin induces the PUM2-dependent incorporation of miR-130a into the exosome membrane [37]. This observation is of great practical importance, as PUM2 may become a new target for anticancer therapy. Although the role of PUM2 in carcinogenesis is widely known, its function in the formation of exosomes modulating cancer cells’ response to cisplatin needs to be thoroughly understood [50,51].

Exosomal lncRNAs are also actively involved in various cancer cells acquiring resistance to cisplatin [38,39,40,41,42]. It should be emphasized that lncRNAs are competitive endogenous RNAs (ceRNAs), meaning that they can bind to miRs through partial complementarity, reducing their level and activity [52]. This regulates the expression of messenger RNAs (mRNAs) [52,53]. For this reason, lncRNAs are often called the molecular “sponge” or “decoy” of miRs [53]. Therefore, later in this manuscript, the functions of exosomal lncRNAs are, if pertinent, referred to as lncRNA/miR/mRNA networks. All the lncRNAs described below act as oncogenes in various types of cancer [54,55,56,57,58]. Therefore, it is justified to study their relationship with resistance to chemotherapy, particularly if they can be transported through exosomes to cytotoxic-sensitive cancer cells.

Li et al. demonstrated that lncRNA urothelial carcinoma-associated 1 (UCA1) is essential for the resistance of ovarian cancer cells to cisplatin, both in vitro and in vivo [38]. Increased expression of lncRNA UCA1 was detected in the serum exosomes of cisplatin-resistant ovarian cancer patients [38]. In the context of resistance to chemotherapeutic agents, this lncRNA shows three critical actions both in vitro and in vivo: (1) it promotes the proliferation of cancer cells; (2) it inhibits their apoptosis; and (3) it reduces cisplatin-induced cytotoxicity [38]. At the core of the molecular mechanism of these changes is that lncRNA UCA1 negatively affects the expression of miR-143, which in turn is a modulator of FOS-like 2, AP-1 transcription factor subunit (FOSL2) expression in ovarian cancer cells [38]. A growing body of evidence suggests a cardinal role for FOSL2 in cancer, especially metastasis [59,60]. High expression of FOSL2 has been confirmed in several types of cancer, including colon cancer [61], breast cancer [62], ovarian cancer [63], liver cancer [64], and osteosarcoma [65]. Therefore, the observation suggests that therapy interfering with the lncRNA UCA1/miR-143/FOSL2 axis could offer a new line of treatment for cisplatin-resistant cancers [38]. Exosomal lncRNA HNF1A antisense RNA 1 (HNF1A-AS1) is another example of this group of nucleic acids that, by decreasing miR-34b expression, promotes tuftelin 1 (TUFT1) expression, thus contributing to the acquisition of cervical cancer cell resistance to cisplatin by enhancing cancer cell proliferation and inhibiting apoptosis [39]. One universal concept that has emerged from previous studies is that TUFT1 promotes cancer development and progression through different signaling pathways [66,67,68,69,70]. High expression of this protein is associated with poor prognosis in several types of cancer, including thyroid cancer [70], liver cancer [69,71], pancreatic cancer [66], gastric cancer [67], lung cancer [67], and breast cancer [67,72]. From this perspective, taking into account the enormous commitment of TUFT1 to carcinogenesis and its related processes, the lncRNA HNF1A-AS1/miR-34b/TUFT1 axis is a tempting exemplification of continuing research into targeted therapy aimed at inhibiting the release and transport of exosomes with high lncRNA HNF1A-AS1 expression [39]. Gastric cancer (GC) is another type of neoplasm in which cells acquire resistance to cisplatin through their ability to “pick up” exosomes containing the lncRNA HOXA transcript at the distal tip (HOTTIP) [40]. The exosomal fraction of lncRNA HOTTIP serves as a ceRNA for miR-218, regulating high-mobility group A1 (HMGA1) expression [40]. Exosome-induced overexpression of HMGA1 causes a number of changes in cancer cells, including the intensification of proliferation, migration, and invasion, and changes in the activity of tissue architecture maintenance proteins [40]. Simply put, the suppression of E-cadherin expression and the increased expression of N-cadherin and vimentin suggest that tumor cells can uptake HMGA1 regulatory exosomes and undergo an immensely active epithelial–mesenchymal transition (EMT) [40]. This observation supports the well-known data, which show that HMGA1, an oncofetal protein, is responsible for the aggressive properties of many cancer cells, leading to poor prognoses for patients in the course of the disease [73,74,75]. Therefore, the regulation of this axis may potentially be used in the therapy of cancer patients. Additionally, TAM-derived exosomes further contribute to developing resistance to cisplatin in GC [42]. GC cells can retrieve exosomes from M2-polarized macrophages expressing lncRNA colorectal neoplasia differentially expressed (CRNDE) [42]. This leads to neural precursor cells expressing developmentally downregulated protein 4-1 (NEDD4-1)-mediated phosphatase and tensin homolog deleted on chromosome 10 (PTEN) ubiquitination, and, consequently, a reduction in PTEN levels and the acquisition of resistance to cisplatin [42]. As in previous studies, exosomes increased the survival of cancer cells and their proliferation while inhibiting apoptosis [42]. The authors further confirmed these results in a mouse model, which demonstrated gastric tumor growth upon exosomes expressing lncRNA CRNDE [42]. The described mechanism is enthralling because it not only reveals how cells of the tumor microenvironment modulate cisplatin resistance but also demonstrates faultlessly how exosomes via lncRNA CRNDE suppress PTEN expression, known as a tumor suppressor [76,77,78,79].

The mechanism of cisplatin resistance, with exosomal lncRNA participation, has also been observed in tongue squamous cell carcinoma [41]. Wang et al. demonstrated that exosomal lncRNA HEIH, by acting as a ceRNA for miR-3619-5p, increases the expression of hepatoma-derived growth factor (HDGF), which leads to the acquisition of cisplatin resistance by cancer cells [41]. Exosomal lncRNA HEIH stimulates the proliferation of cancer cells and inhibits their apoptosis [41]. HDGF is also an oncogene whose high expression in liver cancer [80], lung cancer [81], and gastric cancer [82] is associated with unfavorable patient outcomes [80,81,82]. HDGF regulates the proliferation, angiogenesis, and apoptosis of cancer cells [83,84]; thus, one therapeutic option may be to use antagonists of exosomal lncRNA HEIH.

As can be seen in the findings mentioned earlier, the action of exosomal lncRNA predominantly unbalances the miR–mRNA axis by acting as a sponge for the miR. The upregulation of mRNA expression of several oncogenes, including FOSL2, TUFT1, HMGA1, and HDGF [38,39,40,41], most often leads to the acquisition of cisplatin resistance, while increasing the proliferation of cancer cells and reducing their apoptosis. The results of experiments on cell cultures have been confirmed in studies on animal models and in studies conducted on cancer patients. Exosomes with high expression of lncRNA UCA1, lncRNA HNF1A-AS1, lncRNA HOTTIP, lncRNA HEIH, and lncRNA CRNDE increase the size of tumors in vivo and modulate patients’ lack of response to the applied chemotherapeutic agents [38,39,40,41].

The latest achievements in molecular biology allow us to assess the role of exosomal circRNAs in cancer resistance to cisplatin treatment. This form of nucleic acid is primarily responsible for controlling parental gene expression through various mechanisms, including miR sponges [85], which is particularly important in understanding the role of exosomal circRNAs in cancer cells acquiring cisplatin resistance. As with lncRNA, circRNA disrupts the miR-mRNA axis by decreasing miR and consequently increasing the mRNA expression of several oncogenes, leading to the acquisition of resistance to cisplatin. Research on exosomal circRNAs as modulators of cisplatin resistance has been overwhelmingly focused on NSCLC [44,45,46]. Three different forms of exosomal circRNA are responsible for the resistance of NSCLC cells to this drug, namely hsa_circ_0014235 [44], circ_0008928 [45], and circ_0076305 [46]. They share a common mechanism of action (mentioned above) and constitute the sponge of miRs. Cyclin-dependent kinase 4 (CDK4) is an oncogene that is particularly important for controlling the cell cycle and has been demonstrated in many types of cancer [86,87]. CDK4 and CDK6 inhibitors are used in treating patients with hormone receptor–positive breast cancers [88,89]. Therefore, it is clinically warranted to influence the higher level of expression controlling these proteins, i.e., the use of the hsa_circ_0014235 inhibitor. A study by Xu et al. proved it to be a modulator of CDK4 expression in vitro and in vivo [44]. The authors showed that exosomal hsa_circ_0014235, by inhibiting miR-520a-5p, promotes CDK4 expression, thus contributing to several changes, including not only NSCLC-cell resistance to cisplatin but also cell proliferation, migration, and invasion, which is reflected in increased tumor growth in a mouse model [44]. A similar model of action was described for the case of circ_0008928 [45]. The expression of exosomal circ_0008928 was significantly higher in the blood of NSCLC patients with cisplatin resistance compared with the group of patients sensitive to this chemotherapeutic agent [45]. A detailed analysis showed that exosomal circ_0008928 is a sponge of miR-488 and thus increases the expression of hexokinase 2 (HK2) [45]. Interestingly, from a practical point of view, the inhibition of circ_0008928 increases the sensitivity of cancer cells to cisplatin [45]. HK2, associated initially with glucose metabolism, regulates tumor cell growth, proliferation, metastasis, and apoptosis [90,91,92,93,94]. Silencing its expression may sensitize cancer cells to chemotherapy and radiotherapy [95,96]; hence, the potential inhibition of the interaction of exosomal circ_0008928 with miR-488 may also be helpful in treating cisplatin-resistant cancers. More recently, Wang et al. clearly illustrated how exosomal circ_0076305 enhances adenosine triphosphate (ATP)-binding cassette subfamily C member 1 (ABCC1; also called multidrug resistance-associated protein 1, MRP-1) expression by inhibiting miR-186-5p, leading to increased resistance of NSCLC cells to cisplatin [46]. High expression of exosomal circ_007630 was observed in serum samples obtained from lung cancer patients, and this nucleic acid promoted resistance to cisplatin in the culture of NSCLC cells and in a mouse model [46]. ABCC1/MRP1 determines the ineffectiveness of therapy with several anticancer drugs [97,98]. Its involvement in this process has been known for many years [99,100,101,102]. It is, therefore, not surprising that its expression is modulated by exosomes, given that these types of EVs intensify the chemoresistance of cancer cells with such ferocity.

CircRNAs are also involved in cisplatin resistance in non-NSCLC tumor types, such as epithelial ovarian cancer (EOC) [47], esophageal cancer [48], and gastric adenocarcinoma (GAC) [49]. Naturally, the regulation of resistance to this chemotherapeutic agent in these tumors occurs through sponging miRs. Increased expression of the exosomal circular forkhead box protein P1 (circFoxp1) in patients with EOC has been observed [47]. EOC patients with high serum expression of exosomal circFoxp1 developed resistance to cisplatin, which was also associated with shorter overall survival (OS) and disease-free survival (DFS) than patients with low circFoxp1 expression. Most notably, meticulous analysis proved that circFoxp1 on exosomes can inhibit miR-22 and miR-150-3p, leading to increased expression of two genes, CCAAT enhancer binding protein gamma (CEBPG) and formin-like 3 (FMNL3), which may affect the resistance of cancer cells to cisplatin [47]. Still, the exact mechanism of these genes’ involvement in chemoresistance remains unknown. Nonetheless, it is acknowledged that both genes can induce tumor formation and progression in various types of cancer. CEBPG is involved in the pathogenesis of conditions such as acute myeloid leukemia, esophageal cancer, and lung cancer [103,104,105]. Similar properties are attributed to FMNL3 [106,107], a high expression of which is associated with poor prognosis in patients with colorectal carcinoma, melanoma, or tongue cancer [108,109,110,111]. Moreover, exosomal circ_0000337 enhances the cisplatin resistance of esophageal cancer cells by increasing Janus kinase 2 (JAK2) expression due to miR-377-3p inhibition [48]. Cisplatin-sensitive cells have the ability to acquire resistance by taking up these exosomes, which results in cancer cell growth and metastasis in vitro and in vivo [48]. Finally, exosomes in GAC are characterized by increased expression of circ_0000260, which suppresses the expression of miR-129-5p [49]. This process leads to an increase in the expression of matrix metalloproteinase 11 (MMP11), which functions as an oncogene [49]. The authors indicated that circ_0000260 enhances the proliferation, migration, invasion, and adhesion of cancer cells, as well as tumor growth, in a mouse model [49]. Tumor tissues and exosomes isolated from the serum samples of cisplatin-resistant GAC patients showed high circ_0000260 expression [49]. However, we surmise that these results should be interpreted with a certain degree of caution, as MMP11 is, on the one hand, an oncogene [112,113,114]; on the other hand, it can inhibit proliferation and metastasis in advanced forms of cancers [115]. These studies clearly demonstrate the need for continued efforts to develop a precise therapy that can modulate the expression of circRNAs in exosomes.

Another representative of platinum-containing chemotherapeutic agents to which exosomal ncRNAs modulate the resistance of cancer cells is oxaliplatin (OXA). In this context, researchers have primarily focused on circRNAs [116,117] and miRs [118,119], although single studies have also identified the role of lncRNAs [120] in acquired resistance to oxaliplatin.

Research on the role of exosomal miRs in oxaliplatin resistance has focused substantially on the programmed cell death (PDCD) family. Previously, an extensive range of interactions between miRs and these proteins was demonstrated, responsible for the suppression of the expression of PDCDs in various clinical conditions and, interestingly, in non-cancerous conditions [121]. PDCD4 and PDCD10, described below, play a key role in inhibiting the proliferation and differentiation of cancer cells and enhancing their apoptosis [122,123,124,125,126]. Exosomal miR-46146 is an active participant in colorectal cancer (CRC) cells that develop oxaliplatin resistance [118]. Drug-sensitive CRC cells become resistant through miR-46146-dependent suppression of PDCD10 expression by the uptake of exosomes derived from resistant cells [118]. An intriguing observation was made by Ning et al., showing that exosomal miR-208b released by CRC cells inhibits the expression of PDCD4 in CD4+ T cells and promotes Treg expansion [119]. According to the authors, high levels of miR-208b in the serum are associated with poor clinical outcomes of CRC in patients undergoing FOLFOX (oxaliplatin with l-leucovorin and 5-fluorouracil [5-FU]) therapy [119]. Additionally, the exosomal form of miR-208b enhances tumor growth in the murine model [119]. These results also indicate that resistance to chemotherapeutic agents can be modulated by the influence of neoplastic exosomes on other non-cancerous cells, initially not playing a role in this phenomenon. On the other side of the coin, non-cancerous cells, such as CAFs, can also modulate neoplastic resistance to oxaliplatin via exosomes. Such a process was described for CRC cells, in which CAFs-exosomes with high lncRNA colorectal cancer-associated (CCAL) expression activated the Wnt/β-catenin signaling pathway through direct interaction with mRNA stabilizing human antigen R (HuR; also known as embryonic lethal vision-like protein 1, ELAVL1), thus contributing to the development of the chemoresistance of CRC cells to oxaliplatin [120]. The authors of this study confirmed that cancer cells could take up exosomes from their microenvironment, which modulate their phenotype through the high expression of ncRNA [120]. In this case, CRC cells collected exosomal lncRNA CCAL derived from CAFs [120]. This led to an increase in β-catenin mRNA and protein levels through HuR, manifested in the development of resistance not only to oxaliplatin but also to 5-fluorouracil in both cell cultures and animal models [120].

Sponging miRs by exosomal circRNAs promotes resistance to oxaliplatin [116,117]. This phenomenon has been described in CRC cells [116] and GC cells, among others [117]. In CRC cells, the contribution of exosomal hsa_circ_0005963 (ciRS-122) to oxaliplatin resistance has been proven [116]. By inhibiting the expression of miR-122, this exosomal circRNA leads to the upregulation of the M2 isoform of pyruvate kinase (PKM2) and the resistance of CRC cells to oxaliplatin [116]. Exosomes released from oxaliplatin-resistant cells were readily ingurgitated by sensitive cells, prompting escalation of glucose uptake and synthesis of lactate and ATP [116]. PKM2 exerts several oncogenic effects on cancer cells [127,128,129]. It has been confirmed that PKM2 induces tumor growth and metastasis by controlling cell metabolism and is an active nuclear transcription factor in many pro-cancerogenic signaling pathways [127,128,129]. Exosomal hsa_circ_0005963 also increases glycolysis and drug resistance in vivo; hence, the potential use of its inhibitor may eliminate the growing problem of oxaliplatin’s ineffectiveness in patients diagnosed with CRC [116]. Finally, by sponging miR-515-5p, exosomal circ_0032821 regulates SRY-box transcription factor 9 (SOX9) expression, which is manifested in the resistance of GC cells to oxaliplatin [117]. In the study described, exosomes acted in a way similar to the mechanisms previously indicated by other authors: sensitive cancer cells became resistant to oxaliplatin by accumulating exosomes with high circ_0032821 expression and by actively regulating the miR-515-5p/SOX9 axis [117]. SOX9 has strong oncogenic properties associated with the growth and metastasis of several types of cancer [130,131,132,133,134,135]. Hence, modulation of its activity at the exosomal level may also positively affect counteracting resistance to platinum-containing chemotherapeutic agents.

Table 1 illustrates how exosomal ncRNAs modulate platinum-based chemotherapy resistance in divergent types of cancer. Despite multifarious mechanisms of action by exosomal ncRNAs at the molecular level, phenotypically, they lead preponderantly to increased proliferation of neoplastic cells, which enhances their survival rate. The extended metastatic properties of cancer cells and decreased apoptosis are also a result of the influence of exosomal ncRNAs. These phenomena lead to increased resistance to chemotherapeutic agents. Animal models have shown that exosomal ncRNAs exacerbate tumor growth and lead to clinically noticeable resistance to platinum-based antineoplastic drugs in humans.

### 3.2. Resistance to Alkylating Agents

Among this group of chemotherapeutic agents, cancer cells’ resistance to temozolomide (TMZ), which develops involving exosomal ncRNAs, is the most systematic research subject [136,137,138,139,140]. Since temozolomide is the main line of therapy for high-risk gliomas [141], these studies have investigated the resistance of this type of tumor to temozolomide [136,137,138,139,140]. The process involves exosomal miRs [136], lnRNAs [137,138], and circRNAs [139,140]. As with the previously described chemotherapeutic agents, the most common mechanism is miR blocking by lncRNA or circRNA, although exosomes that overexpress specific miRs are also involved in the acquisition of temozolomide resistance. An example is miR-25-3p, the expression of which is upregulated in exosomes present in cultures of the temozolomide-resistant glioblastoma cell line (A172R) and in the blood of patients treated with this drug [136]. Exosomal miR-25-3p knocks down the F-box and WD repeat domain-containing-7 (FBXW7), a tumor suppressor, leading to glioblastoma cells’ resistance to temozolomide by enhancing the expression of c-Myc and cyclin E, which are known for their oncogenic properties [136]. Thus, the conclusion is that miR-25-3p transported by exosomes disturbs the proteasome-mediated degradation of oncoproteins c-Myc and cyclin E, which are the natural substrates of FBXW7 [142,143]. It does not come as a surprise that the authors reported increased tumor size in mice treated with miR-25-3p. High serum miR-25-3p levels were also a determining factor in the failure of temozolomide treatment [136].

In gliomas, neoplastic cells can also become resistant to temozolomide due to exosomal lncRNAs, such as lncRNA SBF2 antisense RNA 1 (lncRNA SBF2-AS1) [137] and lncRNA temozolomide-associated lncRNA in glioblastoma recurrence (lncRNA TALC) [138]. In the first case, lncRNA SBF2-AS1, a known oncogene, is transported by exosomes and acts as ceRNA for miR-151a-3p, which overexpresses X-ray repair cross-complementing 4 (XRCC4). The consequence of this process is the enhancement of DNA double-strand break (DSB) repair, which plays an essential role in developing resistance to temozolomide [137]. Very recently, Li et al. presented impressive results showing that drug-resistant glioblastoma cells release exosomes with lncRNA TALC expression, which alters the properties of the microglia and causes M2 polarization [138]. LncRNA TALC in these cells activates the enolase 1/p38 mitogen-activated protein kinase/myocyte enhancer factor 2C (ENO1/p38 MAPK/MEF2C) pathway, resulting in complement component **5** (C5) over-synthesis [138]. Next, C5 promotes resistance to temozolomide by enhancing DNA damage repair (DDR) in cells previously sensitive to this chemotherapeutic agent [138]. This study could prove to be a milestone in the treatment of refractory gliomas by inhibiting these signaling pathways. A recent Chinese group described how heparanase, by enhancing the release of hsa_circ_0042003-rich exosomes, promotes the resistance of glioma cells to temozolomide [139]. However, the authors did not describe the precise mechanism through which hsa_circ_0042003 modulates resistance to this chemotherapeutic agent [139]. Although the role of hsa_circ_0042003 is not fully understood, heparanase has been incessantly associated with the process of tumor formation and growth and with poor prognoses in cancer patients [144,145,146,147]. Temozolomide resistance is also modulated by the effect of exosomal circRNA homeodomain-interacting protein kinase 3 (circ-HIPK3) on the miR-421/zinc finger protein of the cerebellum 5 (ZIC5) axis [140]. Circ-HIPK3 sponges miR-421, thus causing ZIC5 overexpression, which can drive tumor progression and drug resistance [140]. These in vitro results were validated in a mouse model with a smaller tumor size and weight, documented after silencing circ-HIPK3 [140]. ZIC5 is assiduously involved in promoting carcinogenesis [148,149,150,151]. Hence, the discovery and characterization of the axis, as mentioned earlier, could be a new way to treat recurrent high-grade glioblastoma.

Table 2 illustrates how exosomal ncRNAs modulate temozolomide-based chemotherapy resistance in glioblastoma. Different groups of researchers [136,137,138,139,140] came to similar conclusions that enhancing tumor cell proliferation and decreasing their apoptosis caused by exosomal ncRNAs determined in vivo intensive tumor growth and resistance to temozolomide in patients diagnosed with glioblastoma. Clinically, patients with high serum and/or exosomal expression of miR-25-3p, lncRNA SBF2-AS1, lncRNA TALC, hsa_circ_0042003, and circ-HIPK3 may have a poor prognosis and more significant mortality [136,137,138,139,140]. The first conclusion from these studies is that the future of individual anti-glioblastoma therapy may lie in drugs interfering with specific molecular axes controlled by exosomal ncRNAs. The second is the potential practical application of exosomal ncRNAs in the search for a patient refractory to temozolomide treatment.

### 3.3. Resistance to Antimetabolite Agents

Representatives of antimetabolites, the anti-tumor activity of 5-fluorouracil (5-FU) and that of gemcitabine (GEM), are modulated by exosomal ncRNAs [152,153,154]. Mao et al. conducted an experiment using two cell lines of lung cancer: (1) a 5-fluorouracil-resistant cell line and (2) a cell line susceptible to this chemotherapeutic agent [152]. They demonstrated that the first cell line, through exosomes with high expression of the lncRNA forkhead box D3 antisense RNA 1 (lncRNA FOXD3-AS1), led the second cell line to acquire resistance to 5-fluorouracil, primarily by inhibiting apoptosis [152]. This oncogenic mechanism is based on the overexpression of ELAVL1 (HuR) and activation of the phosphatidylinositol 3-kinase (PI3K)/protein kinase B (AKT) pathway by exosomal lncRNA FOXD3-AS1 [152]. This is another well-documented example of how the use of lncRNA inhibitors may be crucial in successful cancer chemotherapy. Moreover, it has been reported that exosomal lncRNA CCAL, with the involvement of ELAVL1, modulates resistance to oxaliplatin and 5-fluorouracil [120]. Both studies proved the importance of ELAVL1 in modulating the chemoresistance of cancer cells [120,152].

CRC cells sensitive to 5-fluorouracil become resistant to it by downregulating miR-217 and miR-485-3p through the action of exosomal circRNA_0000338 [153]. The fundamental role of circRNA_0000338 in the burgeoning 5-fluorouracil resistance of CRC cells was confirmed in three stages of the experiment: in CRC cell culture, in a mouse model, and in CRC patients [153]. In this study, however, the authors did not identify the direct target of both miRs, which is key to the precise determination of the mechanism of CRC cell resistance to 5-fluorouracil and the possible practical application of the obtained results [153]. Despite this, previous studies have shown that high expression levels of exosomal circRNA_0000338 may be a marker for predicting CRC resistance to chemotherapeutic agents [155].

Furthermore, gemcitabine resistance in pancreatic cancer (PC) cells is modulated by exosomal circZNF91 [154]. Gemcitabine-resistant cells under hypoxic conditions release exosomes with high circZNF91 expression, and they are transported to normoxic PC cells sensitive to this drug, contributing to deacetylase sirtuin1 (SIRT1) overexpression through the inhibition of miR-23b-3p [154]. In turn, the overexpression of SIRT1 stabilizes the hypoxia-inducible factor 1-alpha (HIF-1α) protein, leading to glycolysis reinforcement in cancer cells [154]. PC cells previously sensitive to gemcitabine, by taking up exosomes derived from hypoxic PC cells, increase proliferation in vitro and tumor growth in vivo [154]. Thus, exosomal ncRNA controls cancer cell metabolism by increasing glycolysis, a process that supplies cancer cells with enormous amounts of energy for proliferation and metastasis.

Table 3 illustrates how exosomal ncRNAs modulate antimetabolite-based chemotherapy resistance in divergent types of cancer. As with the previously discussed groups of anticancer drugs, in the case of antimetabolites, exosomal ncRNAs increase the proliferation of cancer cells and inhibit their apoptosis, effectively reducing their sensitivity to chemotherapeutic agents. In addition, tumor growth in murine models and the failure of chemotherapy in cancer patients may be associated with the high expression of exosomal ncRNAs.

## 4. Exosomes as Chemosensitivity Mediators

Although most relevant studies have investigated the role of exosomes in resistance to chemotherapeutic agents, single studies have also shown a significant role for these EVs and their associated ncRNAs in the promotion of cancer cells susceptible to chemotherapy agents. They also revealed that exosomes themselves could potentially be used as a standalone treatment or as treatments integrated into a chemotherapeutic regimen [156,157,158,159,160,161]. The literature review shows that exosomal miRs [158,160,161] and circRNAs [157,159] have these properties and that drug resistance may be reduced when cisplatin [157,158], oxaliplatin [159], temozolomide [160], and docetaxel [161] are used.

First, extremely interesting results have been presented by an international team showing a link between miR-126 transported by endothelial (human umbilical vein endothelial cells, HUVECs) exosomes and the development of malignant mesothelioma (MM) [156]. In vitro, exosomal miR-126, depending on the research model, time, and dose, may have different effects on different components of the stroma tumor [156]. Briefly, reduced angiogenesis and tumor growth were induced by decreased expression of miR-126 in fibroblasts and increased miR-126 expression in endothelial cells in an miR-126-sensitive environment, which is associated with modulation of vascular endothelial growth factor (VEGF), EGF-like domain multiple 7 (EGFL7), and insulin receptor substrate 1 (IRS1) expression [156]. This is another study demonstrating that exosomal ncRNAs can induce changes in tumor microenvironment cells with the potential practical use of endothelial exosomes with high miR-126 expression in the treatment of MM [156]. One study also showed that exosomal miR-199a-3p enhances the chemosensitivity of hepatocellular carcinoma (HCC) cells to cisplatin. However, in the course of working on the current manuscript, the study was retracted by the editors. We do not, therefore, discuss those results in detail [162].

In addition, the exosomal circRNA sponge for miR-7 (ciRS-7, Cdr1as) can potentially inhibit the cisplatin resistance of ovarian cancer cells by inhibiting miR-1270 and consequently enhancing suppressor of cancer cell invasion (SCAI) expression [157]. In the cell culture and in the mouse model, cicrRNA Cdr1as inhibited proliferation, stimulated apoptosis of tumor cells, and then decreased the weight and volume of the tumor. Analysis of serum samples obtained from patients with cisplatin resistance additionally showed a decrease in exosomal cicrRNA Cdr1as expression [157]. Therefore, exosomes expressing cicrRNA Cdr1as may be a potential marker of clinically noticeable resistant cisplatin [157]. Indeed, other authors have also suggested such a role for cicrRNA Cdr1as [163,164]; however, the question of what the function is for exosomes in transport and the active use of circRNA in other types of cancer remains open.

Cisplatin-resistant GC cells become sensitive to this chemotherapeutic agent via the exosomal transport of miR-107 [158]. MiR-107 downregulates the expression of high mobility group A2 (HMGA2) and inhibits the HMGA2/mammalian target of the rapamycin (mTOR)/P-glycoprotein 1 (P-gp) pathway in resistant GC cells, effectively reducing their ability to proliferate [158]. Interestingly, the authors confirmed that chemotherapeutic agent-resistant cells could take up exosomes from cells sensitive to these drugs, dramatically changing their response to therapy [158]. This observation has practical implications for using exosomes as carriers, either for chemotherapeutic agents or independent drugs, as previous studies have shown the opposite. That is, most of the studies we have cited have demonstrated that the chemotherapeutic agent-sensitive cells take up exosomes derived from the chemotherapeutic agent-resistant tumor cells. Nevertheless, these results [158] should be revised in vivo and ideally in clinical studies. Recently, Xu et al. suggested that the exosomal circRNA FBXW7, by acting as a molecular sponge on miR-18b-5p, leads to an increase in CRC cell apoptosis, inhibition of EMT, and suppression of oxaliplatin efflux, thus contributing to a significant increase in the sensitivity of CRC cells to this drug [159]. CRC cells captured exosomes derived from the human fetal colon epithelial cell line with the expression of circRNA FBXW7 [159]. This inhibited their proliferation in vitro and in vivo [159]. This study clearly proves that exosomes released by physiological cells can reduce the neoplastic capacity and, thus, the invasiveness of tumor cells.

Another type of microRNA, miR-151a, increases glioblastoma cells’ sensitivity to temozolomide through exosomal transport [160]. This is due to the reduction of XRCC4 expression in recipient cells and the inhibition of DSB in them [160]. Those glioblastoma patients with low exosomal expression of miR-151a in the cerebrospinal fluid, but not serum samples, had significantly lower OS compared to patients with high expression of miR-151a on the exosome surface [160]. In turn, the results published in 2020 show that miR-200c is released by normal tongue epithelial cells (NTECs) and transferred to tongue squamous cell carcinoma (TSCC) cells by exosomes [161]. The mechanism of action for exosomal miR-200c is tubulin beta 3 Class III (TUBB3) and protein phosphatase 2 scaffold subunit Abeta (PPP2R1B) suppression, which decreases the migration, invasion, and motility of cancer cells [161]. At the same time, exosomal miR-200c increases the apoptosis of cancer cells, which is additionally confirmed by the smaller tumor sizes observed in vivo in a mouse model [161]. We want to point out that, according to the information on the journal website (access: 23 August 2022), this article is currently undergoing investigation. Therefore, the results should be interpreted with caution. It must be acknowledged that another manuscript on the roles fulfilled by exosomal ncRNAs in oxaliplatin chemosensitivity was retracted by the editor in chief of Molecular Cancer [165]. This demonstrates the need for careful interpretation of data by other authors analyzing papers on the role of exosomes in modulating the chemotherapeutic agent response.

Table 4 illustrates how exosomal ncRNAs modulate sensitivity to different types of chemotherapeutic agents in divergent types of cancers. Although much of the research on the role of exosomal ncRNAs has focused on them as activators of chemoresistance, it has been elegantly explained that these structures can reduce resistance to chemotherapeutic agents. By inhibiting the expression of oncogenes, such as HMGA2 or XRCC4, through miRs [158,160] and inhibiting miRs with oncogenic properties, such as miR-1270 or miR-18b-5p, through specific circRNAs [157,159], exosomes change the phenotype of neoplastic cells from cytostatically resistant to sensitive. This is manifested by a reduction in their proliferation and an increase in apoptosis, as well as a decrease in the metastatic potential or inhibition of the release of drugs from inside the cells.

## 5. Conclusions and Perspectives

**Figure 1:**
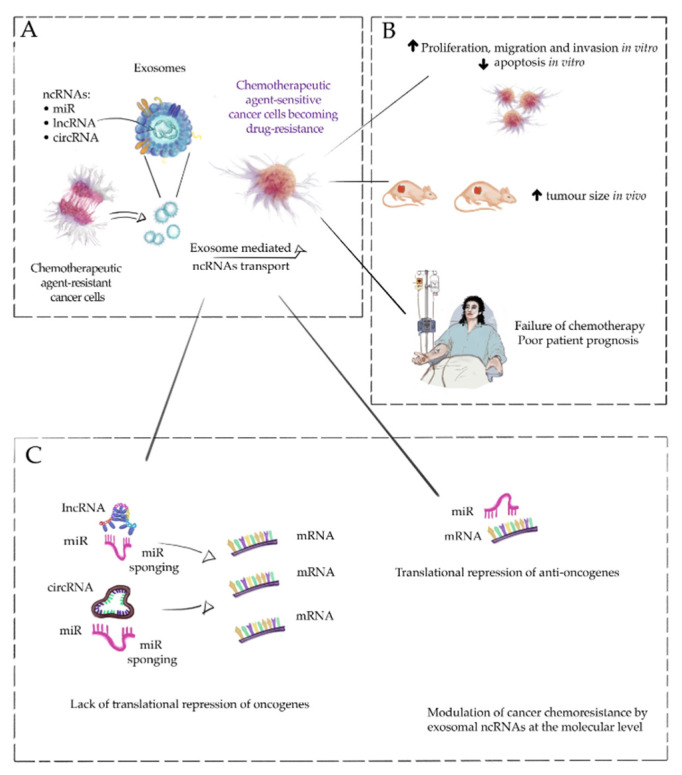
A simplified role of exosomal non-coding RNAs (ncRNAs) in developing cancer cells’ resistance to chemotherapeutic agents. Chemotherapeutic agent-sensitive cancer cells may become drug-resistant through exosomal transport of ncRNAs. Chemotherapy-resistant cancer cells can release exosomes with high expression of ncRNAs, which can be taken up by chemotherapeutic-sensitive cancer cells, changing the properties of the cells and making them chemotherapeutic resistant (**A**). The primary mechanism responsible for this phenomenon is sponging microRNAs (miRs) by exosomal long non-coding RNAs (lncRNAs) and circular RNAs (circRNAs) and, consequently, increasing the expression of oncogenes responsible for the acquisition of chemoresistance (**B**). The two main consequences of these changes at the cellular level are increased proliferation and the inhibition of cancer cell apoptosis. Therefore, tumor growth in vivo and the ineffectiveness of chemotherapeutic agent therapy in oncological patients are associated with increased survival and invasiveness of tumor cells (**C**). Targeted cancer therapy may eventually be based on interactions in the lncRNA/circRNA-miR-mRNA axis.

To make a long story short, our narrative review clearly and in a very detailed way described the exosomes and, in fact, ncRNAs associated with them increase the resistance of cancer cells to chemotherapeutic agents. The primary mechanism by which chemoresistance is modulated is miR sponging by lncRNAs and circRNAs, disrupting the miR-mRNA axis, which leads to the overexpression of oncogenes. These processes systematically lead to the acquisition of chemoresistance, manifested by an increased proliferation of tumor cells, a lack of apoptosis, and the inhibition of the active efflux of drugs from the cancer cells. Most of the studies analyzed in our review also showed that exosomes significantly increase the size of tumors in vivo. From the clinical point of view of the studies we reviewed, a fundamental conclusion is that high expression of exosomal ncRNAs is associated with developing resistance to chemotherapeutic agents in cancer patients; hence, the potential practical application of measuring this expression in predicting patient response to treatment. To facilitate the understanding of these complex molecular mechanisms, we present them in a simplified manner in Figure 1. Understanding these mechanisms and precisely following the axis of ncRNA-miR-mRNA is crucial for the future use of exosome inhibitors in cancer therapy. Based on the analyzed research and our observations [14,30,166,167], it must be noted that both small and large EVs are the future of personalized medicine, including not only in the diagnosis and prognosis of cancer patients but also as a treatment strategy. Such an application of our knowledge of exosomes can rely on two potential activities: (1) inhibiting the release of exosomes [168] and (2) using exosomes as a platform for chemotherapeutic agent transport [169,170] and as a ‘freighter’ for therapeutic RNAs and peptides [171]. Here, advanced techniques of biomedical engineering come to our aid [172,173,174,175]. EVs modified by these methods may successfully become part of oncological therapy [172,173,174,175]. However, to win this war against cancer, which has been conducted for many years, research into its clinical application must be carried out. This is an opportunity that modern evidence-based medicine cannot miss.

## Figures and Tables

**Table 1:** Detailed analysis of exosomal non-coding RNAs (ncRNAs) mediated resistance to platinum-based anticancer therapy.

Type of Cancer	Anticancer Agent	EXO Cellular Source	EXO Cargo	Targeted Regulatory Network	Main In Vitro ncRNAs/EXO Effect	Main In Vivo ncRNAs/EXO Effect	Ref.
Esophageal cancer	Cisplatin	TE-1/DDP	miR-193	TFAP2C	(1) Inhibition of cisplatin-induced cancer cell cycle arrest (2) Inhibition of cancer cell apoptosis	(1) Increasing the tumor size in a mouse model	[36]
Non-small cell lung cancer	Cisplatin	CAFs	miR-130a	ND	(1) Increasing the cancer cell survival rate	(1) Increasing the tumor size in a mouse mode	[37]
Ovarian cancer	Cisplatin	ND*	lncRNA UCA1	miR-143/FOSL2	(1) Increasing the cancer cell proliferation (2) Inhibition of cancer cell apoptosis (3) Decreasing cisplatin-induced cytotoxicity	(1) Increasing the tumor size in a mouse mode	[38]
Cervical cancer	Cisplatin	HeLa/DDP	lncRNA HNF1A-AS1	miR-34b/TUFT1	(1) Increasing the cancer cell proliferation (2) Inhibition of the cancer cell apoptosis	(1) Increasing the tumor size in a mouse mode	[39]
Gastric cancer	Cisplatin	MGC-803/DDP MKN-45/DDP	lncRNA HOTTIP	miR-218/HMGA1	(1) Increasing the cancer cell proliferation, migration, and invasion	(1) Chemotherapy failure of GC patients was associated with high lncRNA HOTTIP expression	[40]
Tongue squamous cell carcinoma	Cisplatin	SCC4/DDP	lncRNA HEIH	miR-3619-5p/HDGF	(1) Increasing the cancer cell proliferation (2) Inhibition of cancer cell apoptosis	ND	[41]
Gastric cancer	Cisplatin	TAMs	lncRNA CRNDE	NEDD4-1/PTEN	(1) Increasing the cancer cell survival rate and proliferation (2) Inhibition of cancer cell apoptosis	(1) Increasing the tumor size in a mouse mode	[42]
Non-small cell lung cancer	Cisplatin	ND *	hsa_circ_0014235	miR-520a-5p/CDK4	(1) Increasing the cancer cell proliferation, migration, and invasion	(1) Increasing the tumor size in a mouse mode	[44]
Non-small cell lung cancer	Cisplatin	ND *	circ_0008928	miR-488/HK2	(1) Increasing the cancer cell proliferation, migration, and invasion	(1) Chemotherapy failure of NSCLC patients was associated with high circ_0008928 expression	[45]
Non-small cell lung cancer	Cisplatin	ND *	circ_0076305	miR-186-5p/ABCC1	(1) Increasing the cancer cell survival rate	(1) Increasing the tumor size in a mouse model	[46]
Epithelial ovarian cancer	Cisplatin	ND *	circFoxp1	miR-22/miR-150-3p/CEBPG/FMNL3	(1) Increasing the cancer cell survival rate and proliferation	(1) Increasing the tumor size in a mouse mode (2) Chemotherapy failure of EOC patients was associated with high circFoxp1 expression	[47]
Esophageal cancer	Cisplatin	EC9706/DDP KYSE30/DDP	circ_0000337	miR-377-3p/JAK2	(1) Increasing the cancer cell proliferation, migration, and invasion (2) Inhibition of cancer cell apoptosis	(1) Increasing the tumor size in a mouse model	[48]
Gastric adenocarcinoma	Cisplatin	ND *	circ_0000260	miR-129-5p/MMP11	(1) Increasing the cancer cell proliferation, migration, and invasion (2) Inhibition of cancer cell apoptosis	(1) Increasing the tumor size in a mouse mode (2) Chemotherapy failure of GAC patients was associated with high circ_0000260 expression	[49]
Colorectal cancer	Oxaliplatin	SW480/OXA	hsa_circ_0005963 (ciRS-122)	miR-122/PKM2	(1) Increasing the cancer cell survival rate (2) Inhibition of cancer cell apoptosis	(1) Increasing the tumor size in a mouse model	[116]
Gastric cancer	Oxaliplatin	HGC27/OXA AGS/OXA	circ_0032821	miR-515-5p/SOX9	(1) Increasing the cancer cell proliferation, migration, and invasion	(1) Increasing the tumor size in a mouse model	[117]
Colorectal cancer	Oxaliplatin	HCT116/OXA HT29/OXA	miR-46146	PDCD10	(1) Increasing the cancer cell proliferation (2) Inhibition of cancer cell apoptosis	ND	[118]
Colorectal cancer	Oxaliplatin	SW480/OXA	miR-208b	PDCD4	(1) Tregs expansion	(1) Increasing the tumor size in a mouse model	[119]
Colorectal cancer	Oxaliplatin	CAFs	lncRNA CCAL	HuR/ Wnt/β-catenin	(1) Increasing the cancer cell survival rate (2) Inhibition of cancer cell apoptosis	(1) Increasing the tumor size in a mouse model	[120]

* Exosomes were extracted from patients’ serum samples. Abbreviations:ABCC1 = adenosine triphosphate (ATP)-binding cassette subfamily C member 1, AGS/DDP = cisplatin-resistant gastric adenocarcinoma cell line, AGS/OXA = oxaliplatin-resistant gastric adenocarcinoma cell line, CAFs = cancer-associated fibroblasts, CDK4 = cyclin-dependent kinase 4, CEBPG = CCAAT enhancer binding protein gamma, circFoxp1 = circular forkhead box protein P1, DDP = cisplatin (cis-diamminedichloroplatinum), EC9706/DDP = cisplatin-resistant esophageal squamous cell line, EOC = epithelial ovarian cancer, EXO = exosomes, FMNL3 = formin-like 3, FOSL2 = FOS like 2, AP-1 transcription factor subunit, GAC = gastric adenocarcinoma, GC = gastric cancer, HCT116/OXA = oxaliplatin-resistant colon carcinoma cell line, HDGF = hepatoma-derived growth factor, HeLa/DDP = cisplatin-resistant HeLa cell line, HGC27/DDP = cisplatin-resistant gastric carcinoma cell line, HGC27/OXA = cisplatin-resistant gastric carcinoma cell line, HK2 = hexokinase 2, HMGA1 = high-mobility group A1 gene, HT29/OXA = oxaliplatin-resistant colon adenocarcinoma cell line, HuR = human antigen R, JAK2 = Janus kinase 2, KYSE30/DDP = cisplatin-resistant esophageal squamous cell line, lncRNA = long non-coding RNA, lncRNA CCAL = lncRNA colorectal cancer-associated, lncRNA CRNDE = lncRNA colorectal neoplasia differentially expressed, lncRNA HEIH = lncRNA high expression in hepatocellular carcinoma, lncRNA HNF1A-AS1 = lncRNA HNF1A antisense RNA 1, lncRNA HOTTIP = lncRNA HOXA transcript at the distal tip, lncRNA UCA1 = lncRNA urothelial carcinoma-associated 1, MGC-803/DDP = cisplatin-resistant gastric mucinous adenocarcinoma cell line, MKN-45/DDP = cisplatin-resistant gastric adenocarcinoma cell line, MMP11 = matrix metalloproteinase 11, ncRNAs = non-coding RNAs, ND = not determined, NEDD4-1 = neural precursor cells expressing developmentally downregulated protein 4-1, NSCLC = non-small cell lung cancer, OXA = oxaliplatin, PDCD10 = programmed cell death 10, PDCD4 = programmed cell death 4, PKM2 = M2 isoform of pyruvate kinase, PTEN = phosphatase and tensin homolog deleted on chromosome 10, SCC4/DDP = cisplatin-resistant tongue squamous cell carcinoma cell line, SOX9 = SRY-box transcription factor 9, SW480/OXA = oxaliplatin-resistant colon adenocarcinoma cell line, TAMs = tumor-associated macrophages, TE-1/DDP = cisplatin-resistant esophageal cancer cells, TFAP2C = transcription factor AP-2 gamma, Tregs = regulatory T cells, TUFT1 = tuftelin 1.

**Table 2:** Detailed analysis of exosomal non-coding RNAs (ncRNAs) mediated resistance to alkylating therapy.

Type of Cancer	Anticancer Agent	EXO Cellular Source	EXO Cargo	Targeted Regulatory Network	Main In Vitro ncRNAs/EXO Effect	Main In Vivo ncRNAs/EXO Effect	Ref.
Glioblastoma	Temozolomide	A172/TMZ	miR-25-3p	FBXW7	(1) Increasing the cancer cell proliferation (2) Inhibition of cancer cell apoptosis	(1) Increasing the tumor size in a mouse model (2) Chemotherapy failure of GBM patients was associated with high miR-25-3p expression	[136]
Glioblastoma	Temozolomide	Rec GBM N3T3rd	lncRNA SBF2-AS1	miR-151a-3p/XRCC4	(1) Increasing the cancer cell proliferation (2) Inhibition of cancer cell apoptosis (3) Increasing the DNA damage repair	(1) Increasing the tumor size in a mouse model (2) Chemotherapy failure of GBM patients was associated with high lncRNA SBF2-AS1 expression	[137]
Glioblastoma	Temozolomide	LN229/TMZ	lncRNA TALC	ENO1/p38 MAPK/MEF2C/C5	(1) Inhibition of cancer cell apoptosis (2) Increasing the DNA damage repair	(1) Increasing the tumor size in a mouse model (2) High expression of C5 induced by lncRNA TALC was associated with a poor prognosis in mice and GBM patients	[138]
Glioblastoma	Temozolomide	U251/TMZ	hsa_circ_0042003	Heparanase	(1) Increasing the cancer cell proliferation (2) Inhibition of cancer cell apoptosis	(1) Increasing the tumor size in a mouse model (2) Chemotherapy failure of GBM patients was associated with high hsa_circ_0042003 expression	[139]
Glioblastoma	Temozolomide	A172/TMZ U251/TMZ	circ-HIPK3	miR-421/ZIC5	(1) Increasing the cancer cell proliferation and invasion (2) Inhibition of cancer cell apoptosis	(1) Increasing the tumor size in a mouse model (2) Chemotherapy failure of GBM patients was associated with high circ-HIPK3 expression	[140]

Abbreviations: A172/TMZ = temozolomide-resistant glioblastoma cell line, C5 = complement component **5**, circ-HIPK3 = circRNA homeodomain-interacting protein kinase 3, ENO1 = enolase 1, EXO = exosomes, FBXW7 = F-box and WD repeat domain-containing-7, GBM = glioblastoma, LN229/TMZ = temozolomide-resistant glioblastoma cell line, lncRNA = long non-coding RNA, lncRNA SBF2-AS1 = lncRNA SBF2 antisense RNA 1, lncRNA TALC = lncRNA temozolomide-associated lncRNA in glioblastoma recurrence, MEF2C = myocyte enhancer factor 2C, N3T3rd = temozolomide-resistant glioblastoma cell line, ncRNAs = non-coding RNAs, ND = not determined, p38 MAPK = p38 mitogen-activated protein kinase, Rec GBM = temozolomide-resistant glioblastoma cell line, TMZ = temozolomide, U251/TMZ = temozolomide-resistant astrocytoma cell line, XRCC4 = X-ray repair cross complementing 4, ZIC5 = zinc finger protein of the cerebellum 5.

**Table 3:** Detailed analysis of exosomal non-coding RNAs (ncRNAs) mediated resistance to antimetabolite therapy.

Type of Cancer	Anticancer Agent	EXO Cellular Source	EXO Cargo	Targeted Regulatory Network	Main In Vitro ncRNAs/EXO Effect	Main In Vivo ncRNAs/EXO Effect	Ref.
Lung cancer	5-fluorouracil	A549 SPC-A1	lncRNA FOXD3-AS1	ELAVL1/PI3K/Akt	(1) Increasing the cancer cell proliferation and invasion (2) Inhibition of cancer cell apoptosis	ND *	[152]
Colorectal cancer	5-fluorouracil	SW480/5-FU HCT116/5-FU	circ_0000338	miR-217/miR-485-3p	(1) Increasing the cancer cell proliferation (2) Inhibition of cancer cell apoptosis	(1) Increasing the tumor size in a mouse mode (2) Chemotherapy failure of CRC patients was associated with high circ_0000338 expression	[153]
Pancreatic cancer	Gemcitabine	SW1990 BxPC-3	circZNF91	miR-23b-3p/SIRT1	(1) Increasing the cancer cell proliferation (1) Increasing HIF-1α-dependent glycolysis in cancer cell	(1) Increasing the tumor size in a mouse mode (2) High expression of circZNF91was associated with a poor prognosis in PC patients	[154]

* Although the authors did not define the role of exosomal lncRNA FOXD3-AS1 in vivo, they showed its high expression in tissues collected from lung cancer patients. Abbreviations: 5-FU = 5-fluorouracil, A549 = adenocarcinomic human alveolar basal epithelial cell line, BxPC-3 = pancreatic ductal adenocarcinoma cell line, CRC = colorectal cancer, ELAVL1 = embryonic lethal vision-like protein 1, EXO = exosomes, GEM = gemcitabine, HCT116/5-FU = 5-fluorouracil-resistant colon carcinoma cell line, HIF-1α = hypoxia-inducible factor 1-alpha, lncRNA = long non-coding RNA, lncRNA FOXD3-AS1 = lncRNA forkhead box D3 antisense RNA 1, ncRNAs = non-coding RNAs, ND = not determined, PC = pancreatic cancer, PI3K/Akt = phosphatidylinositol 3-kinase/protein kinase B, SIRT1 = sirtuin 1, SPC-A1 = human lung adenocarcinoma cell line, SW1990 = pancreatic adenocarcinoma cell line, SW480/5-FU = 5-fluorouracil-resistant colon adenocarcinoma cell line.

**Table 4:** Detailed analysis of exosomal non-coding RNAs (ncRNAs) mediated sensitivity to different types of chemotherapeutic agents in divergent types of cancers.

Type of Cancer	Anticancer Agent	EXO Cellular Source	EXO Cargo	Targeted Regulatory Network	Main In Vitro ncRNAs/EXO Effect	Main In Vivo ncRNAs/EXO Effect	Ref.
Ovarian cancer	Cisplatin	ND *	circRNA Cdr1as	miR-1270/SCAI	(1) Inhibiting the cancer cell proliferation and migration (2) Increasing the cancer cell apoptosis	(1) Decreasing the tumor size in a mouse model (2) Chemotherapy failure of OC patients was associated with low circRNA Cdr1as expression	[157]
Gastric cancer	Cisplatin	SGC-7901 MGC-803	miR-107	HMGA2/mTOR/P-gp	(1) Inhibiting the cancer cell proliferation	ND	[158]
Colorectal cancer	Oxaliplatin	FHC	circRNA FBXW7	miR-18b-5p	(1) Inhibiting the cancer cell proliferation migration, and invasion (2) Inhibiting OXA efflux (3) Increasing the cancer cell apoptosis	(1) Decreasing the tumor size in a mouse model (2) Chemotherapy failure of OC patients was associated with low circRNA FBXW7 expression	[159]
Glioblastoma	Temozolomide	GBM cell lines	miR-151a	XRCC4	(1) Inhibiting the cancer cell proliferation (2) Inhibiting the DNA damage repair (3) Increasing the cancer cell apoptosis	1) Decreasing the tumor size in a mouse model (2) Low expression of miR-151a was associated with a poor prognosis in GBM patients	[160]
Tongue squamous cell carcinoma	Docetaxel	NTECs	miR-200c	TUBB3/PPP2R1B	(1) Inhibiting the cancer cell viability, migration, invasion, and motility (2) Inhibiting the DNA damage repair (3) Increasing the cancer cell apoptosis	(1) Decreasing the tumor size in a mouse model	[161]

* Exosomes were extracted from patients’ serum samples. Abbreviations: circRNA = circular RNA, circRNA Cdr1as = circular RNA sponge for miR-7, circRNA FBXW7 = circular RNA F-box and WD repeat domain-containing-7, EXO = exosomes, FHC = human colon epithelial cell line of fetal, GBM = glioblastoma, HMGA2 = high mobility group A2, lncRNA = long non-coding RNA, MGC-803 = gastric mucinous adenocarcinoma cell line, mTOR = mammalian target of rapamycin, ncRNAs = non-coding RNAs, ND = not determined, NTECs = normal tongue epithelial cells, OC = ovarian cancer, OXA = oxaliplatin, P-gp = P-glycoprotein 1, PPP2R1B = protein phosphatase 2 scaffold subunit Abeta, SCAI = suppressor of cancer cell invasion, SGC-7901 = human gastric cancer cell line, TUBB3 = tubulin beta 3 class III, XRCC4 = X-ray repair cross complementing 4.

## Data Availability

Not applicable.
